# Evaluating Perceptions of the CANreduce 2.0 eHealth Intervention for Cannabis Use: Focus Group Study

**DOI:** 10.2196/65025

**Published:** 2025-03-19

**Authors:** Daniel Folch-Sanchez, Maria Pellicer-Roca, María Agustina Sestelo, Paola Zuluaga, Francisco Arias, Pablo Guzmán Cortez, Salma Amechat, Gustavo Gil-Berrozpe, Estefania Lopez Montes, Clara Mercadé, Francina Fonseca, Laia Miquel, Joan I Mestre-Pintó

**Affiliations:** 1 Health and Addictions Research Group, Addictions Unit Psychiatry and Psychology Service, Institut Clínic de Neurociències (ICN) Hospital Clinic Barcelona, Institut d'Investigacions Biomèdiques August Pi i Sunyer (IDIBAPS) Barcelona Spain; 2 Hospital del Mar-Research Institute and Universitat Pompeu Fabra Neuroscience Research Program Addiction Research Group (GRAd) Barcelona Spain; 3 Hospital Universitari Germans Trias i Pujol Unidad de Medicina Interna Badalona Spain; 4 Hospital Universitario 12 de Octubre Instituto de Investigación i+12 Madrid Spain; 5 Hospital Universitario de Navarra Navarra Spain; 6 Parc de Salut Mar Institut de Neuropsiquiatria i Adiccions (INAD) Barcelona Spain; 7 Hospital del Mar Medical Research Institute (IMIM) Barcelona Spain; 8 Department of Medicine and Life Sciences (MELIS) Universitat Pompeu Fabra Barcelona Spain

**Keywords:** addiction, cannabis, drug use disorder, eHealth, digital health intervention, qualitative research, focus groups, user-centered design, user-centered intervention

## Abstract

**Background:**

Cannabis is the most widely used illicit drug, and admissions for cannabis use disorders (CUDs) are increasing globally, posing a significant public health challenge. Despite its negative consequences, a substantial proportion of individuals with problematic use do not seek treatment. In recent years, digital health interventions (DHIs) have emerged as accessible and cost-effective solutions, empowering users to manage their health care. CANreduce is one such eHealth intervention that has demonstrated effectiveness in reducing cannabis use (CU); however, its suboptimal adherence rates underscore the need for strategies to enhance user engagement and motivation.

**Objective:**

This study aims to improve the effectiveness, adherence, and user experience of the Spanish version of CANreduce 2.0 by employing focus groups (FGs) within a user-centered design approach that actively involves both users and professionals.

**Methods:**

Separate FGs were conducted for users and professionals, involving a total of 10 participants. Users were recruited from individuals registered on the CANreduce 2.0 platform and active cannabis users, while professionals comprised addiction specialists familiar with the platform. Each session was held remotely and moderated by 2 interviewers following a semistructured script. Qualitative analysis of the transcripts was performed using MAXQDA software and content analysis methodology to identify key themes related to the acceptability, usability, and utility of CANreduce 2.0.

**Results:**

The qualitative analysis identified 3 main themes, encompassing 15 subcodes. Within the “motivation and awareness” theme, both users (n=6, mean age 31.8 years, SD 4.1 years) and professionals (n=4, mean age 37.25 years, SD 1.71 years) frequently discussed the importance of “motivation” and “problem awareness” as crucial for the success of CANreduce 2.0. In the “guidance and use” theme, the subcode “complement to face-to-face therapy” was the most emphasized. Professionals supported CANreduce 2.0 as a valuable adjunct to in-person therapy, serving as both an educational and monitoring tool, with no objections raised by either group. Lastly, within the “content and design” theme, “information,” “small achievements,” and “personalized content” emerged as key areas for improvement, highlighting the need to enhance motivation and adherence through gamification and tailored content.

**Conclusions:**

Personalization, robust motivational strategies, and an engaging, interactive design are essential for the success of DHIs, particularly in addiction treatment. Collaboration among technology developers, health care professionals, and users should be central to the development process, fostering the cocreation of practical and effective solutions that are responsive to the needs of those seeking treatment. This approach ensures that DHIs are not only functional but also widely accepted and impactful. Insights from this study will inform the ongoing refinement of CANreduce 2.0, enhancing its relevance and effectiveness in addressing CU.

## Introduction

Cannabis is the most widely used drug worldwide, posing a significant problem due to its negative effects on users’ well-being and its broader impact on public health. According to the latest report from the United Nations Office on Drugs and Crime [1], approximately 228 million people consumed cannabis in 2022, with many countries reporting a sustained increase in the prevalence of cannabis use (CU), hospitalizations, and diagnosed psychiatric disorders related to CU.

In Spain, cannabis is the illicit substance with the highest prevalence, particularly among younger individuals. The latest data estimate that 19.1% of individuals under 35 years of age [2] and 21.8% of students aged 14-18 years used cannabis in the past year, with the average age of initiation being 15 years [3]. The National Strategy on Addictions [4] places special emphasis on the normalization of CU among youth and the challenges this poses, given that early onset correlates not only with an increased risk of developing cannabis use disorder (CUD) but also with other mental health conditions. Furthermore, early use is associated with academic and occupational difficulties [1]; involvement in conflicts, fights, or physical aggression [3,5]; and engagement in risky sexual behaviors [3,6].

As a result, the total number of admissions for CU treatment is increasing [2,7], with 93.7% of individuals under 18 years initiating treatment for illicit drug use in Spain doing so for CU [2]. Spain has a well-established, extensive, diversified, and accessible network for addressing drug-related issues. Among the available treatments, motivational interviewing and cognitive behavioral therapies (CBTs) have proven effective in managing substance use disorders (SUDs) as therapeutic and psychoeducational strategies [8-10], both in-person and digitally [11,12].

However, despite the availability and variety of treatments, a significant proportion of problematic cannabis users do not seek help, often due to social stigma, shame, lack of time, or the belief that they can regulate their consumption on their own [13,14]. This issue is particularly pronounced among younger individuals, who not only seek treatment less frequently but also exhibit lower adherence rates [15].

Technological progress in the 21st century is driving the digitalization of everyday life, with screens becoming the primary medium for communication and self-expression among young people [16]. In response to this shift, the development of mobile apps and internet-based interventions for health promotion and treatment has grown steadily over the past 2 decades, offering patients accessible and cost-effective solutions that empower them to take control of their health care journey [17]. In the context of mental health and SUDs, these tools can function as (1) adjuncts to clinical care and (2) strategies for health promotion, enhancing accessibility to treatment.

Multiple meta-analyses have examined the use of digital health interventions (DHIs) for addressing SUDs [18], particularly CUDs [15,19-22]. Evidence from these studies demonstrates a favorable yet modest effect of digital health strategies, with greater efficacy in prevention programs compared with intervention programs. However, both approaches show a lower impact than face-to-face interventions [19], highlighting the need to develop strategies that integrate both modalities [10,21,22].

CANreduce is one of the eHealth tools proven effective in reducing CU. It is a minimally guided, internet-based self-help intervention developed by Schaub et al [23] and has been certified as safe by the European Union, qualifying as a medical device under directives 93/42/EWG and 2007/47/EWG. This interactive online platform consists of 8 modules designed to be completed over 6 weeks, integrating CBT strategies, motivational interviewing, and immediate ecological interventions.

In 2015, the first version of the app proved to be an effective alternative to face-to-face treatment, demonstrating reductions in the frequency and quantity of CU, increased completion of self-help modules, and higher abstention rates, although adherence remained suboptimal [23]. Despite the flexibility DHIs offer, allowing them to be tailored to individual user needs, poor adherence remains a common challenge with this type of tool [17,24]. Adherence is a complex, dynamic process influenced by individual factors such as motivation and self-efficacy, interpersonal elements such as therapeutic alliances and shared decision-making, and therapy-related aspects, including preplanned interventions and medication regimens [25]. Some of these factors—particularly interpersonal elements—are typically absent from digital interventions such as CANreduce, as they generally do not provide clinician support. Effective strategies to address this issue and improve adherence to digital treatments [26], particularly for CU [20], include incorporating support from a mental health professional or coach [27] and using participation reminders [28].

Following the principles of Adherence-Focused Guidance [29], CANreduce 2.0 was developed and tested in a subsequent clinical trial. This new edition incorporated support from either an “e-Coach” or a team without a specific reference individual, with both interventions proving equally effective in reducing CU frequency and mental health symptoms [30]. In 2022, the app was adapted to Spanish and validated for use within the Spanish population. Data from the corresponding clinical trial are currently being analyzed, and its protocol has been previously published [31]. The adaptation included adding videos featuring Laura, the Spanish e-Coach, along with 6 characters of different ages, genders, and backgrounds, illustrating various situations of cannabis abuse and dependence throughout the modules.

The World Health Organization has emphasized the importance of using a user-centered design in the development of digital tools, noting that most people do not re-engage with an app if it does not initially attract them [32]. Previous research has shown that patients will only use new technologies if they are relevant to their health concerns, visually appealing, easy to use, and effective in facilitating behavior change [17]. Increasing patient involvement in the design of DHIs is essential for creating relevant, usable, and effective solutions and has been linked to greater acceptability and motivation for use [33,34], thereby improving user experience and perceived value [35]. Motivation is a critical component of behavior change, helping predict patient abstinence and reductions in substance use. In the context of SUD and AUD treatment, it is a multidimensional, fluid state that enables individuals to resolve their ambivalence about making difficult changes to avoid substance abuse [25,36]. It has consistently been associated with increased treatment adherence and completion, making it a key construct in the Adherence-Focused model [25]. While motivation is intrinsic to patients, interventions can help them identify their reasons and need for change, as well as facilitate the planning required to achieve it [36].

User-centered design is a multiphase process that involves conducting focus groups (FGs) or interviews with users, modifying designs and content based on qualitative findings, and continuously iterating these steps with additional user and professional input [17,35,37]. Qualitative methods enable an in-depth evaluation by uncovering attitudes, perceptions, and beliefs that influence interaction with the tool, while also assessing user needs and preferences related to interface and usability [38,39].

A co-design approach that actively involves users has a significant impact on health outcomes and provides numerous benefits, including improved adherence [25], fidelity, and reach of digital tools [40]. Additionally, it fosters a broader range of ideas, enhances usability [41,42], and increases user satisfaction, support, and enthusiasm for innovation. It also improves decision-making efficiency and strengthens the patient-clinician relationship [17,35].

This study aims to enhance the effectiveness, adherence, and user experience of the Spanish version of CANreduce 2.0 through a user-centered design approach. As the initial phase of a larger research project, its findings on user perspectives will inform subsequent stages to improve the development of a DHI for CU treatment.

## Methods

### Recruitment

Discussion groups were conducted separately for users and professionals. For the users’ FG, participants were recruited from those who had registered on the CANreduce 2.0 platform within the past 2 years. Following established guidelines [43], the initial FG aimed to include 6-12 participants. Twelve individuals were contacted by phone and invited to participate; however, only 3 attended, necessitating a second FG session. The second group also included 3 participants—active cannabis users attending treatment for the first time—who were instructed to use the CANreduce 2.0 platform before participating in the discussions. These participants were recruited through snowball sampling and contacted by phone to schedule the second online FG. The professional FG comprised 4 mental health and addiction specialists with experience in using digital tools. All professionals were members of the Primary Care Network for Addictions (RIAPAd) and were involved in promoting the CANreduce 2.0 improvement study for future implementation in addiction treatment centers. These participants were invited via email.

In total, 3 FG sessions were conducted: 2 for users (n=6) and 1 for professionals (n=4). Eligibility criteria for both groups included regular use of mobile devices and no diagnosis of a severe mental illness. Users were required to actively use the CANreduce 2.0 platform and report current CU, while professionals needed prior familiarity with the platform. Each FG session was facilitated by 2 interviewers (DFS and JIMP) and conducted remotely via Zoom (Zoom Communications, Inc./Qumu Corporation) to accommodate participants’ schedules and geographical constraints. Audio recordings of the sessions were made to facilitate transcription.

### Focus Group Interviews

The categories evaluated during the participatory groups were determined through a deductive analysis of usability guidelines focused on user-centered design and a review of scientific literature on digital health apps. Special consideration was given to strategies for motivating change [44,45].

The FGs aimed to elicit expert and participant feedback on both the content and form of the app (interface and materials), as well as the utility of the intervention (assessment of usage and experience). Specific objectives were established: defining the platform’s target population, clarifying its specific purposes, updating and enriching available content, optimizing interface design, and promoting user motivation and adherence. Based on these objectives, a series of questions were formulated, with slight adaptations in each session depending on whether participants were professionals or users. Each FG session lasted approximately 90 minutes.

Semistructured interview scripts were used, allowing for the inclusion of emerging questions relevant to the stated objectives. The questions were designed to align with the study’s purpose and objectives, with some formulated to broadly explore each category (eg, “Do you think there are any missing topics or areas?”) and others aimed at eliciting specific feedback on particular aspects of the intervention (eg, “Do you think using a mobile app format could improve user adherence to treatment?”). Depending on the depth of information provided in each category, either broad or focused questions were applied. To enhance user motivation within the CANreduce platform, options such as increased reminder frequency and positive reinforcement (eg, congratulatory messages) were explored. These examples served as probes, used only when participants did not provide spontaneous responses, to avoid directing their feedback. Interview guides for the professional and user groups can be found in Multimedia Appendices 1 and 2, respectively, with both having been translated from Spanish to English for publication purposes.

### Statistical Analysis

The qualitative analysis of FG transcriptions was conducted using MAXQDA (VERBI GmbH), a software that enabled the systematic coding and analysis of textual data from user transcripts. The software’s coding functionality was used to assign text segments to various codes, representing different themes relevant to the research.

This analysis followed the framework of content analysis, a methodological approach that involves identifying and defining categories or codes to capture key topics of interest. This approach enabled researchers to efficiently manage qualitative data, ensuring that the findings were grounded in participants’ experiences and perspectives.

### Ethics Approval

The study was approved by the Ethics Committee of CEIm-Parc de Salut Mar/IMIM-Hospital del Mar (approval code 2019/8901/I). Before the FGs, informed consent was obtained from all participants. At the start of each session, participants were briefed on key ethical considerations, including confidentiality and voluntary participation, to ensure an ethical and transparent process. Audio recordings were made to facilitate transcription, with no names recorded to maintain confidentiality. Data were fully anonymized throughout the analysis. As compensation, each participant received a €15 (US $15.63) Amazon voucher. No identifying information about participants is included in the manuscript or multimedia appendices.

## Results

### Demographics

The professional FG included 2 men and 2 women, all with higher education and an average age of 37.25 (SD 1.71) years. Half were psychologists (n=2) and the other half were doctors (n=2). They worked in a detoxification hospitalization unit (n=1), an outpatient addiction center (n=2), and an acute psychiatric hospitalization unit (n=1).

Among users (n=6), the 3 participants who had received online treatment through the platform were Spanish women—2 with technical training and 1 with higher education. The new users (n=3) were all Spanish men with higher education. The mean age of users (n=6) was 31.8 (SD 4.1) years.

Finally, both users and professionals had owned smartphones for more than 10 years, except for 1 professional who had owned a smartphone for 7 years. All participants also possessed at least one other electronic device, such as a laptop or tablet.

### Themes

#### Overview

The codes identified in the qualitative analysis of the transcripts were categorized into 3 main themes: “motivation and awareness,” “guidance and use,” and “content and design,” each containing specific subcodes. For “motivation and awareness,” the subcodes included “user age,” “motivation,” “problem awareness,” and “economic awareness.” Within “guidance and use,” the subcodes comprised “virtual treatment” (further divided into “in favor” and “against”), virtual support from Laura (subdivided into “in favor,” “against,” and the importance of the “therapist’s gender” on the platform), and “complement to in-person therapy” (with “in favor” and “against” subcategories). For the “content and design” theme, the identified subcodes were “app” “notifications,” “information,” “small achievements and gamification,” and the availability of “personalized content.”

A comprehensive scheme of all codes can be found in Multimedia Appendix 3. The transcription of participant responses, organized according to the aforementioned codes, is available in Multimedia Appendix 4. Codes, themes, and transcriptions were translated from Spanish into English solely for publication purposes.

**Figure 1:**
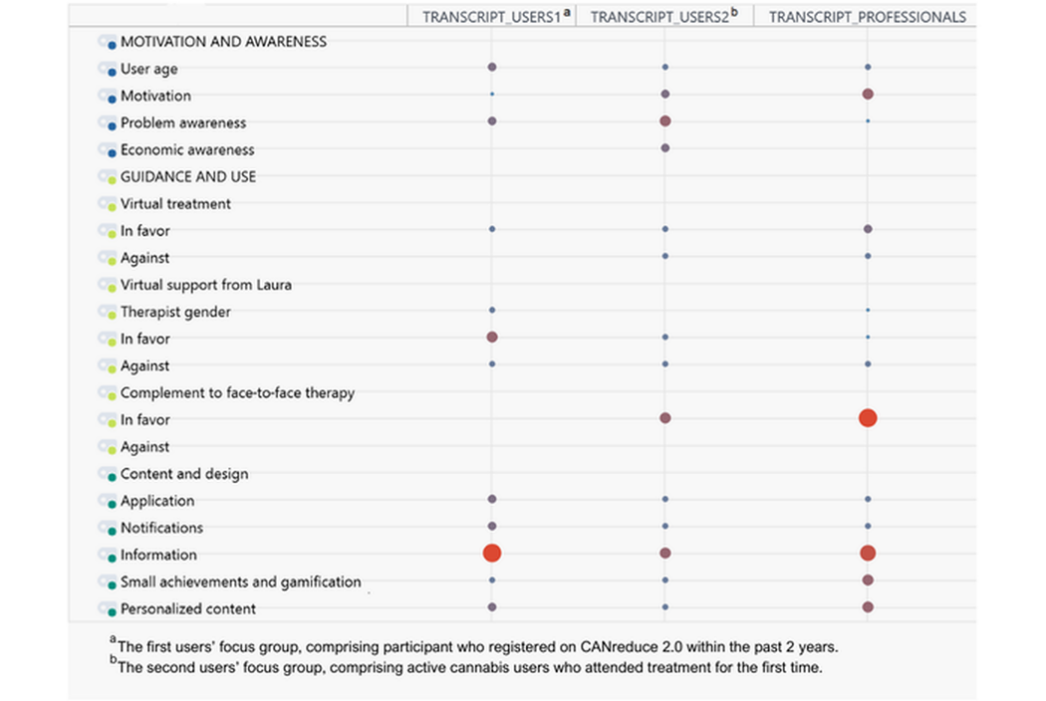
Code distribution across groups.

To analyze the distribution of codes across FG transcripts, a figure from MAXQDA’s Code Matrix Browser was created (Figure 1). Larger symbols in the matrix indicate a higher number of coded segments assigned to a specific code for each group, highlighting key differences and similarities in how users and professionals prioritize different topics.

In the “motivation and awareness” theme, the subcode “user age” was addressed by all groups, with users from the first FG (registered on CANreduce 2.0 within the past 2 years) discussing it slightly more. “Motivation” was mentioned most prominently by professionals, followed by active cannabis users in the second FG (first-time treatment participants) and the first users’ group. Users in the second group prioritized “problem awareness,” discussing it more frequently than users in the first group and professionals, while also being the only group to address “economic awareness.”

In the “guidance and use” theme, all groups discussed the subcode “virtual treatment,” with varying degrees of emphasis. Professionals most frequently highlighted the potential benefits of CANreduce 2.0 as an online treatment for reducing CU but also expressed the greatest concerns about its drawbacks. Active cannabis users in the second FG mentioned some concerns, while users in the first group did not raise objections. The subcode “virtual support from Laura” was addressed equally across all groups, with balanced perspectives on its potential advantages and disadvantages. By contrast, “complement to face-to-face therapy” was primarily explored by professionals, who discussed it extensively and favorably.

In “content and design,” users in the first FG referenced subcodes such as “app” and “notifications” the most, emphasizing the importance of usability and engagement tools. However, “information” was the most discussed subcode within this theme across all groups, reflecting its high priority for both users and professionals. Professionals led the discussion on “small achievements and gamification” and devoted the most attention to “personalized information,” more so than either user group.

#### Motivation and Awareness

##### User Age

Users had mixed opinions on the platform’s target population age. Some felt it was better suited for young people due to its online format and emphasis on less invasive treatment methods, which might appeal to those who have not yet recognized the severity of their consumption habits. Others believed it was more appropriate for adults over 30 years, who are more likely to be aware of their behavior and the consequences of CU.

Professionals noted that the content was designed to reflect young people’s experiences but pointed out some comments they found “slightly uncomfortable” or “potentially sexist.”

##### Motivation

Users emphasized the importance of self-motivation for the platform’s success, noting that without a genuine commitment to reducing or ceasing CU, the provided tools might be ineffective. Some users attributed this to the platform’s inherent characteristics, which they perceived as resembling an “online course” or a “control tool.”

Professionals emphasized the need for the platform to better align with users’ individual goals, noting that it may be less effective in influencing the stage of change, particularly in cases of ambivalence. To address this, they suggested implementing an initial survey or questionnaire to assess the user’s profile, allowing for more tailored interventions.

##### Problem Awareness

Users acknowledged the platform’s effectiveness in increasing awareness of cannabis consumption, helping them identify key moments in the change process, track the frequency and quantity of their use, and recognize its impact on daily life. They perceived the activities as tools that enabled them to “name their feelings” and, by “seeing themselves represented,” become “more aware of their problem.” By articulating their habits, users felt empowered to “reconsider” them and recognize them as problematic. Character testimonies and usage tracking features were seen as facilitators, with the module on managing craving symptoms highlighted as particularly helpful.

##### Economic Awareness

Users noted the significant motivational role of visualizing the economic impact of their cannabis consumption. They observed that the platform lacks this functionality and suggested incorporating tools to calculate cannabis-related expenditures.

#### Guidance and Use

##### Virtual Treatment

Users and professionals identified several benefits of virtual treatment (*in favor*), including greater intimacy and accessibility, particularly for new users or those with moderate cannabis consumption. They noted that the platform enables anonymity, offering a more “private” approach to addressing cannabis abuse. Some professionals viewed virtual treatment as a better first approach, while others emphasized that the platform’s modules appeared designed to address both ends of the consumption spectrum—targeting individuals with SUDs as well as those engaging in recreational use—and could serve as a support tool during the maintenance stage.

Some users and professionals raised concerns (*against*) that virtual treatment lacks the warmth and personalization inherent to in-person interactions, which may be essential for users with severe CU. Professionals argued that DHIs “cannot encompass the full scope of work conducted in face-to-face therapy” and warned that users might “become frustrated if they fail to reduce their use,” potentially deterring them from seeking in-person help. Specifically, they noted that the platform may not be effective for patients with higher dependency consumption patterns or dual pathology.

##### Virtual Support From Laura

Some users mentioned that women are often associated with caregiving roles and suggested that having a female therapist (*therapist gender*) as a guide in the app could be more effective “from a marketing point of view.”

Many users valued the presence (*in favor*) of a humanized name and persona on the platform, as it enhanced engagement and provided a sense of individualized support. They emphasized the importance of “being able to talk to a person or feeling that someone is answering” and expressed a desire for additional features, such as a forum or chat for real-time dialogue, direct contact with someone, or having “Laura personally check in on their progress” during treatment.

Conversely, some users felt that they “do not need anyone to check on their progress.” Additionally, some participants found the online format, including Laura’s support, to be overly impersonal, describing text messages (SMS and email) and online assistance as inherently “cold” (*against*).

##### Complement to In-Person Therapy

Users considered the platform a “starting point for seeking help” and a valuable complement to in-person therapy. They noted that access to cannabis-related information and intake logs could optimize in-person sessions by allowing more time to address “individual and personalized” concerns. To enhance continuity and efficacy in treatment, they emphasized the importance of professionals having access to the information recorded on the platform.

Professionals viewed the platform as a complement rather than a substitute for in-person therapy, recommending its use as an educational and monitoring tool. They highlighted its potential utility for users on therapy waiting lists and its applicability in primary care and various disciplines, such as social work and nursing. The data provided by the platform were seen as beneficial, either by equipping patients with more information to enhance awareness or by serving as a support tool through patient-registered data. However, they noted that the platform currently lacks information on local resources for additional support, which could enhance its effectiveness as a complementary tool.

#### Content and Design

##### App

Users emphasized the difficulty in visualizing the app on mobile devices. They expressed a preference for an app with personalized notifications and tracking features to improve interaction and usability. They mentioned the possibility of receiving “feedback” as a desirable feature.

Professionals suggested that adapting the platform into a mobile app could significantly improve adherence and facilitate regular access.

##### Notifications

Users had varying opinions on the utility of notifications. Some felt that notifications, being common in apps, could serve as reminders to encourage use and improve adherence. Others, however, preferred them to be optional, with one user describing them as feeling “like homework for children.” There was no consensus on the preferred frequency of notifications.

Professionals suggested allowing users to customize the frequency and type of notifications to enhance their usefulness and prevent boredom or annoyance. They also noted that notifications could serve as a means to provide valuable information on accessing alternative forms of treatment, further integrating the virtual platform with in-person care.

##### Information

Users appreciated the information provided but noted that it was occasionally too “dense,” leading to boredom. They suggested incorporating more interactive and visual formats, such as videos instead of text, and simplifying language by reducing technical jargon. While some users found characters illustrating CU experiences helpful, others felt they did not accurately reflect their own situations. Recommendations included adding more information on the negative consequences of CU, stress and anxiety management, improving the visualization of consumption graphs, introducing a search function for quick access to specific information, and incorporating a “panic button” to remind users of their personal goals during moments of craving.

Professionals found the information to be well-written and accessible but agreed with users that it could benefit from being more dynamic and visual. They also emphasized the importance of facilitating navigation to ensure easy access to relevant content, suggesting features such as a search box for specific terms and topics.

##### Small Achievements and Gamification

Users indicated that incorporating gamification elements could enhance the platform’s appeal and motivational impact. They found the forms somewhat boring, with one user noting, “it feels like the platform is an educational course.” They also suggested the inclusion of encouraging messages that acknowledge their progress or improvements in their health status.

Professionals suggested implementing progressive goals and rewards to foster user engagement and satisfaction. However, there was no consensus on whether modules should be unlocked sequentially as users progress.

##### Personalized Content

Users expressed a strong preference for more personalized content tailored to their individual situations and needs, believing this would enhance the platform’s relevance and effectiveness. They highlighted the usefulness of sections for recording personal “excuses” and setting individualized goals.

Professionals stressed the importance of customizing content to align with users’ stages in the change process and personal goals. They also suggested simplifying content based on users’ cognitive or attentional capacities to enhance accessibility and comprehension.

### Overview of Research Findings to Define Next Steps

To incorporate users’ suggestions into the app, specific recommendations were collected based on each code and its subcodes (Textbox 1). Moving forward, these insights will be integrated into the development process to ensure the resulting DHI remains user-centered.

**Recommendations for enhancing CANreduce 2.0 based on focus group feedback from professionals and users for each subcode.**

1. Motivation and awareness
  - User age: personalize according to life stages.
  - Problem awareness: adjust to the level of self-consciousness, including specific information for the precontemplation stage. It could be set with an initial user profile survey.
  - Motivation: include reward systems and more information about the negative consequences of cannabis use.
  - Economic awareness: add functionalities to visualize the economic and social costs of consumption. It could be used as part of the gamification function.
2. Guidance and use
  - Virtual treatment: include a forum or chat to facilitate real-time dialogue.
  - Virtual support from Laura: personalize interaction.
  - Complement to face-to-face therapy: implement its use with patients on waiting lists for therapy and in primary care, particularly with disciplines such as social work and nursing. Allow and promote professionals access to the information registered by their patients.
3. Content and design
  - App: improve usability in smartphones, introduce interactive content (improve form design, introduce a search box).
  - Notifications: implement notifications as reminders and motivational messages, allowing users to choose to receive them or not, the type of notifications, and the frequency.
  - Information: replace part of the text-based content with videos or other visual formats. Include a panic button to be used during cravings, to give quick access to personal reminders of self-goals, pros/cons, or benefits expected.
  - Small achievements and gamification: add motivational messages, incorporate progressive goals, and show the health benefits of the goals achieved.
  - Personalized content: tailor the content to meet users’ needs, goals, and capabilities.

**Figure 2:**
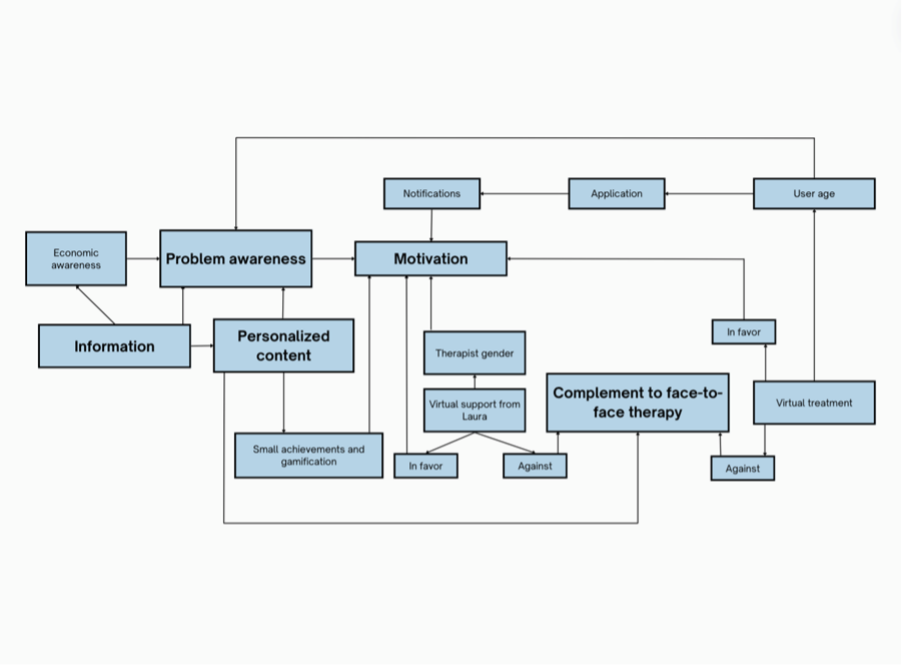
Key themes for increasing usability and adherence in CANreduce 2.0.

To further guide the enhancement of usability and adherence, a relational schema of key themes was developed to illustrate the interconnections and dynamics among the identified codes (Figure 2). This schema visualizes how codes interact and influence each other, highlights emerging patterns, and supports the interpretation of qualitative data. Additionally, it serves as a foundation for generating new hypotheses for future research.

The relational schema of codes was developed through a rigorous qualitative analysis of the FG transcripts. The process began with a detailed transcription of all sessions, followed by the coding of meaning units. The resulting codes were then grouped into broad themes representing related concepts. Subsequently, relationships among these codes were analyzed using concept mapping techniques. The final schema was validated by subject matter experts.

Figure 2 visually represents the interrelations among the key themes identified in the analysis—motivation and awareness, guidance and use, and content and design—highlighting core elements that enhance user experience and adherence. Notably, utilizing the “information” provided within the platform and integrating “personalized content” can strengthen “problem awareness,” which, in turn, increases user “motivation.” Additionally, personalized content is perceived as valuable for using the platform as a “complement to face-to-face therapy,” aiming to promote sustained engagement and adherence to the CANreduce 2.0 program and overall treatment for CUD.

## Discussion

### Principal Findings

This study evaluates the perceptions of users and professionals regarding the utility and potential effectiveness of CANreduce 2.0 in reducing CU, as well as their suggestions for improving its usability and, consequently, adherence to online treatment.

Regarding the main themes evaluated in the FGs, several conclusions can be drawn from the results. First, both users and professionals agreed that CANreduce 2.0 could serve as a valuable complement to in-person therapy. Second, most participants emphasized that the information provided in the modules significantly increased problem awareness and, consequently, sustained motivation to quit or reduce CU. Finally, to enhance adherence to virtual treatment, both users and professionals suggested incorporating notifications, gamification elements, and personalized features to adapt the app to individual users.

### Motivation and Awareness

The findings reveal significant discrepancies in perceptions regarding the optimal age for using the platform. Some users viewed the digital format as more suitable for younger individuals, whereas others believed middle-aged adults, who may have greater insight into their consumption habits, could benefit more. While DHIs are generally assumed to be preferred by younger populations, research remains inconclusive [46]. Studies on web-based interventions have shown that recruited participants tend to be older than those typically treated in specialized addiction centers [47], and dropout rates are higher among younger individuals [48]. Given these uncertainties, it is recommended that the intervention be adapted to allow for personalized modules and support features tailored to specific life stages, maturity levels, and self-awareness, ensuring that each age group finds the tool relevant and effective.

Regarding motivation and problem awareness, it is noteworthy that during recruitment for the CANreduce 2.0 study, the Google search advertisement appeared more than 250 times per day, with an average of 19.6 individuals clicking on the app link. Given the rising demand for CU treatment [2,7] and the persistent lack of access for many individuals [15], a freely available tool discoverable through a simple internet search could serve as a highly valuable resource for those seeking help with their consumption but unable or unwilling to engage in traditional interventions. Digital tools offer several advantages, including anonymity, continuous availability, elimination of physical travel, and rapid accessibility [15,27], which were highlighted in the FGs as key benefits of digital health treatments, particularly CANreduce 2.0.

As for motivation, both users and professionals identified it as a crucial factor in achieving changes in consumption patterns. When asked about the potential effectiveness of the intervention in reducing CU, participants closely linked the tool’s utility to the user’s intrinsic motivation and problem awareness at the time of engagement. Interviewed professionals suggested that CANreduce 2.0 is suitable for individuals in both active consumption and maintenance phases of abstinence, though it may be less effective for those who remain ambivalent. Consequently, they proposed incorporating information specifically tailored for individuals in the precontemplation stage. According to the Stages of Change Model, individuals in this initial stage are not yet considering modifying their consumption habits in the foreseeable future, as they lack full awareness of the associated problems [36]. The first step in addressing an SUD is for the individual to recognize their own problematic behavior and consider making a change. Users acknowledged that the tool effectively assesses their CU, raises awareness of its health consequences, and records instances of excessive consumption, which could positively impact self-efficacy [49]. However, the results also highlighted the need for additional strategies to sustain meaningful and continuous motivation. Proposed features for integration included a reward or incentive system to reinforce progress, expanded information on the negative consequences of CU and withdrawal syndrome, and interactive tools, such as simulations or expense calculators, to help users visualize the economic and social costs of their consumption.

### Guidance and Use

There is substantial evidence demonstrating the impact of mental health apps on the general population, including individuals without symptoms. These apps have shown potential as cost-effective, accessible, and low-intensity intervention tools for those unable or unwilling to receive standard treatment [26]. However, adherence to treatment remains a significant challenge in digital mental health and addiction interventions [50]. Evidence suggests that one strategy to improve adherence is the provision of user-centered guidance.

The randomized clinical trial of CANreduce 2.0 conducted in the Swiss population demonstrated the app’s efficacy in reducing the frequency of use, severity of dependence, and anxiety symptoms. However, it did not find significant differences between support provided by a personalized figure and that offered by a nonindividualized support team [30].

To thoroughly examine these findings, study participants were asked about their experiences and perceptions regarding the virtual support provided by Laura, the therapist integrated into the treatment process through introductory videos for each module and direct messaging. Opinions were diverse. Supporters of virtual assistance emphasized the importance of “humanizing” the experience, suggesting that a forum or a space for more personalized guidance could be beneficial. They noted that personalized interaction, even in a virtual format, fostered a sense of connection and support. Conversely, some participants expressed disinterest in virtual interaction, preferring to forgo this type of communication. Professionals echoed this concern, criticizing virtual support as impersonal and advocating for in-person accompaniment, which they believed offered a more effective and empathetic form of support.

Despite these perceptions, “Dr. Google” and “ChatGPT” remain among the most frequently used tools for medical and treatment inquiries, with people becoming increasingly familiar with their use [51-53]. Mental health apps are often accessed through internet searches and social media [54], which presents certain risks due to the generally poor quality of online information [55] and the subjective, often biased nature of its evaluation [56,57].

Among the interviewed user groups, there was strong support for using the platform as a virtual treatment. They highlighted its potential as an ecological intervention and recognized its advantages in providing an accessible and flexible treatment option. However, professionals expressed reservations about its effectiveness as a standalone tool, particularly for individuals with severe dependence or dual disorders. Although evidence suggests that individuals with more severe symptoms are more likely to complete such treatments [58], professionals questioned whether the app was adequate for these cases. Nonetheless, both users and professionals acknowledged the platform’s value as an adjunct to in-person therapy. With its educational and monitoring features, the information provided could serve as an effective support tool for professionals. This perspective aligns with existing evidence showing that DHIs can enhance treatment effectiveness and support abstinence [59,60]. Integrating the platform with traditional therapy could maximize its strengths in providing ongoing support and monitoring while ensuring comprehensive care for individuals with more complex needs.

### Content and Design

The CANreduce 2.0 app implements a program based on motivational interviewing and CBT, designed to increase disorder awareness, confront users with their consumption problem, and encourage commitment to modifying their CU patterns. This combination of methods, along with its modular structure, aligns with evidence suggesting that apps with such designs demonstrate greater effectiveness [30].

The proposal to register CU habits was well received by FG participants, including both users and professionals. This feature was recognized for its utility in patient monitoring and its potential to promote positive behaviors while reducing negative ones [61]. Additionally, participants suggested incorporating gamification elements and small achievements to enhance motivation and engagement, as these features could provide further incentives for users to stay committed to their treatment and actively engage with the platform’s resources.

A limitation of this DHI, as highlighted during FG discussions, is its lack of adaptation to each user’s unique case. The customization of digital health apps, particularly in terms of usability, remains an underexplored area compared with commercially driven digital apps [39]. User-centered design extends beyond aesthetics and interface dynamics to encompass adaptation to the demographic characteristics, mental models, and psychological needs of the target population [9]. The flow of app use—spanning user experience and interface design—is a fundamental aspect of digital apps. FG research serves as a key method for evaluating these elements [40], and this study provided insights into the perceptions of both users and professionals. Further exploration of individual user expectations and goals could enhance personalization and improve the care process [62].

Users expressed a preference for a mobile app featuring personalized notifications and interactive content, aligning with research suggesting that such features could enhance engagement and adherence [63]. The importance of tailoring content to individual needs and characteristics was a recurrent theme in discussions.

Regarding the user interface, participants highlighted challenges in accessing the app from mobile devices, as it was initially designed as a web-based platform before being adapted for smartphones. They found the predominantly text-based content burdensome and suggested incorporating videos or other visual formats to improve engagement [15]. Additionally, enhancing the visualization of data graphs was identified as a necessary improvement.

### Next Steps

To improve adherence and usability in CANreduce 2.0, feedback from users and professionals (Textbox 1) will be integrated into the DHI. Specifically, motivation and awareness will be enhanced by incorporating a reward system and a visualization tool that highlights the individual economic costs of consumption (expense calculator). Additional content will be included to provide deeper insights into the negative consequences of CU and withdrawal syndrome, along with tailored elements for individuals in the precontemplation stage, as identified through an initial user profiling survey. Motivation and readiness to change are critical mechanisms in mental health therapeutic adherence [25], particularly in the precontemplation stage, where they are most affected. Moreover, motivation is a dynamic process that fluctuates across the different stages of change and varies in intensity over time [25,36]. Thus, integrating specific modules and interventions that address these fluctuations is crucial to maintaining adherence and preventing potential dropouts.

For enhanced guidance and usability, CANreduce 2.0 will incorporate a voluntary forum feature to facilitate peer support and interaction. Additionally, its potential application in addiction treatment centers will be further explored. Consistent with existing evidence [59,60], both users and professionals have recognized CANreduce 2.0 as a valuable complement to face-to-face therapy, particularly for highly motivated individuals. Consequently, future DHI development will prioritize its integration as a therapeutic adjunct, especially for patients on waiting lists and in primary care. Additionally, the feasibility of enabling professionals to access and track patient data will be explored to further enhance clinical support.

Design and content improvements will prioritize usability and accessibility. CANreduce 2.0 will be redesigned as a web application to enhance functionality on smartphones and improve interface interactivity. Planned optimizations include gamification elements, such as the previously mentioned reward system, individual progressive goal tracking, a search tool, optional notifications, and increased visual content to replace text-heavy sections.

Following the implementation of these features, additional FGs will be conducted to gather updated user and professional perspectives, ensuring the continuation of the iterative co-design process. These sessions will also explore areas with limited consensus, such as the optimal user age and necessary adaptations to tailor the intervention for different life stages and levels of self-awareness.

Poor adherence remains a major barrier to implementing DHIs [17,24] and CUD treatments, particularly among younger populations [15]. Motivation, a key construct in the Adherence-Focused model, has been linked to increased treatment completion [25]. As highlighted by FG participants, interventions such as CANreduce 2.0 may heavily depend on user motivation while also supporting individuals in identifying their reasons for change and facilitating planning [36]. For future studies on DHIs, motivation should be carefully considered, as both users and professionals emphasized—consistent with existing literature—that it significantly impacts adherence and, consequently, the intervention’s potential success or failure.

Furthermore, the differences observed between groups in Figure 1 emphasize the importance of addressing varying priorities and perspectives when designing therapeutic tools or interventions. Misalignment in certain areas highlights the need to strengthen communication and cocreation processes. Bridging this gap could help ensure that all stakeholders recognize the value of key features and their potential to enhance therapeutic outcomes. Incorporating diverse perspectives throughout the design and development process is crucial for creating interventions that are both effective and widely adopted. A participatory approach—engaging both professionals and users—could align priorities, foster shared ownership of solutions, and enhance long-term impact and sustainability.

### Limitations

This study has limitations, including the small group sizes and the fact that some surveyed users were not highly familiar with the app. However, evidence suggests that such unfamiliarity can provide valuable insights into user experience evaluation [64]. While the FG strategy employed in the protocol is effective for exploring motivations, experiences, and subjective perceptions in-depth, it is inherently limited in its level of evidence, as it lacks statistical representativeness and does not allow for the measurement of phenomena [38].

The use of a co-design model often presents challenges, including the significant time, effort, and financial resources required, as well as the complexity of balancing diverse perspectives, which can complicate decision-making [65-67]. Another common barrier is the generalizability of outputs; as with many co-design studies, our small group sizes may not fully represent the broader user population. Additionally, involving affiliated professionals in the process may influence project direction and introduce potential biases [67]. Despite these challenges, co-design offers substantial benefits, primarily by ensuring that the intervention aligns with end user needs. This approach allows us to directly integrate user perspectives into the design, enhancing relevance, acceptability, and usability [65,68]. Moreover, perceived value or buy-in is widely recognized as a key enabler of co-design, fostering sustained stakeholder engagement and ultimately supporting adoption [67]. The co-design process also generated innovative ideas for improving CANreduce 2.0—such as incorporating notifications and small achievements—making the intervention more applicable to real-life situations and increasing the likelihood of successful integration [66,68].

### Conclusions

The findings of this study highlight the complexity of designing effective DHIs for CU. While technology offers unique opportunities to engage users, the results emphasize the need to address the diverse needs of different demographic groups and severity levels to enhance motivation and adherence. Personalization, combined with robust motivational strategies and an interactive design, appears to be critical for the success of such platforms. Furthermore, collaboration among technology developers, health care professionals, and end users is essential to creating digital solutions that are both practical and effective.

Advancing the design of DHIs that effectively address substance use in innovative and engaging ways remains a challenge in medical informatics and mental health. Integrating user perspectives and values into the design process is essential for enhancing the acceptability, usability, and utility of these tools. For CANreduce 2.0, the insights gained from this study will guide the implementation of necessary improvements, refining both the app’s content and interactive features.

## Appendix

Multimedia Appendix 1 Interview guide for professionals.

Multimedia Appendix 2 Interview guide for users.

Multimedia Appendix 3 Overview of codes from the qualitative analysis of the transcripts.

Multimedia Appendix 4 Qualitative results with codes from the interview's transcriptions.

## Abbreviations

CBT: cognitive behavioral therapy
CU: cannabis use
CUD: cannabis use disorder
DHI: digital health intervention
FG: focus group
SUD: substance use disorder

## Data Availability

All data generated or analyzed during this study are included in this published article and its multimedia appendices.
