# Supplementary material for: Evaluating Perceptions of the CANreduce 2.0 eHealth Intervention for Cannabis Use: Focus Group Study
Source: J Med Internet Res. 2025 Mar 19;27:e65025. doi: 10.2196/65025 (PMC11966080; doi:10.2196/65025)
Supplement: Multimedia Appendix 1 [file jmir_v27i1e65025_app1.docx]

## Multimedia Appendix 1: Interview guide for professionals

| **Objectives** | **Questions** |
| --- | --- |
| **Define the target population (cannabis consumers)** | What kind of patients do you think this platform can be most useful for?  Do you think it is aimed at people who want to access treatment for the first time and prefer an initial online approach? |
| **Define the intended use of the platform** | Do you think this tool can replace in-person treatment, or would you solely recommend it as a complementary aid? What type of professionals could benefit from having this tool as a complement to the in-person treatment they provide? |
| **Update content** | Do you think there are any missing topics or areas?  Do you think any of the current topics or areas are unnecessary?  Do you consider any content to be overly detailed?  Do you consider any content to be insufficiently detailed? |
| **Improve platform design** | What are your thoughts on the platform's design?  How did you find the navigation experience on the website?  Do you think the information is presented in a dynamic and interactive way, or do you find the platform rather monotonous?  Do you believe the information is well integrated?  Do you find the platform visually appealing? |
| **Improve user motivation and adherence** | Regarding the human guidance within the platform, would you suggest any modifications (such as increasing the frequency of reminders or incorporating positive reinforcements like congratulatory messages, etc.) to improve user motivation?  What specific needs concerning treatment do you believe patients with cannabis use problems have?  Do you think using a mobile application format could improve user adherence to treatment? |
