# Supplementary material for: Evaluating Perceptions of the CANreduce 2.0 eHealth Intervention for Cannabis Use: Focus Group Study
Source: J Med Internet Res. 2025 Mar 19;27:e65025. doi: 10.2196/65025 (PMC11966080; doi:10.2196/65025)
Supplement: Multimedia Appendix 2 [file jmir_v27i1e65025_app2.docx]

## Multimedia Appendix 2: Interview guide for users

| **Objectives** | **Questions** |
| --- | --- |
| **Define the target population (cannabis consumers)** | Who do you think can benefit from CANreduce? People with high consumption or low consumption? People who have never visited a therapist and are looking for an initial contact? |
| **Define the intended use of the platform** | Do you believe this is a useful and adequate treatment to quit or reduce cannabis use?  Do you think it is better to complement this online intervention with in-person treatment by a therapist? |
| **Update content** | Is the content easy to understand?  Do you consider that the platform or any specific content provides too much or too little information? |
| **Improve platform design** | What are your thoughts on the platform's design?  How did you find the navigation experience on the website?  Do you think the information is presented in a dynamic and interactive way, or do you find the platform rather monotonous?  Do you believe the information is well integrated?  Do you find the platform visually appealing? |
| **Improve user motivation and adherence** | Why do people who see the platform online decide not to use it?  What reasons might lead people to stop using the platform?  Do you think the constant participation of a real therapist within the platform can help improve user motivation?  What specific needs concerning treatment do you believe patients with cannabis use problems have?  Do you think using a mobile application format could improve user adherence to treatment? |
