# Supplementary material for: Evaluating Perceptions of the CANreduce 2.0 eHealth Intervention for Cannabis Use: Focus Group Study
Source: J Med Internet Res. 2025 Mar 19;27:e65025. doi: 10.2196/65025 (PMC11966080; doi:10.2196/65025)
Supplement: Multimedia Appendix 3 [file jmir_v27i1e65025_app3.docx]

## Multimedia Appendix 3: Overview of codes from the qualitative analysis of the transcripts

| **Codes** | **Subcodes** |
| --- | --- |
| Motivation and awareness | User age |
|  | Motivation |
|  | Problem awareness |
|  | Economic awareness |
| Guidance and use | Virtual treatment   - In favor - Against |
|  | Virtual support from Laura   - Therapist’s gender - In favor - Against |
|  | - Complement to in-person therapy |
| Content and design | Application |
|  | Notifications |
|  | Information |
|  | Small achievements and gamification |
|  | Personalized content |
