# Supplementary material for: Evaluating Perceptions of the CANreduce 2.0 eHealth Intervention for Cannabis Use: Focus Group Study
Source: J Med Internet Res. 2025 Mar 19;27:e65025. doi: 10.2196/65025 (PMC11966080; doi:10.2196/65025)
Supplement: Multimedia Appendix 4 [file jmir_v27i1e65025_app4.docx]

## Multimedia Appendix 4: qualitative results with codes from the interview’s transcriptions

| **Codes** | **End-user remarks** | **Clinicians remarks** |
| --- | --- | --- |
| **Motivation and awareness** |  |  |
| **User age** | - "There is no age in which it can be more useful" - "I think it is more focused on older ages, starting from 30, because at 20 you aren’t aware of how much it can harm you." - "I agree that you have to have done some prior reflection." - "The audience that can use the platform is a young one. In that sense, I think it is very well suited for a young person who has started out using cannabis or already has a consolidated consumption. Whether they consume a lot or not, I think it can be very useful for both cases unless it's a very critical case where the help would be minimal." - “Many of the people who don’t access hospital care are young people who might be a good fit for this because they might not realize the seriousness of their problem. This approach, which could be seen as a less invasive treatment option, might be more appealing to them." | - "Looking at the testimonials made me wonder if it was more for teenagers or for adults, but then I thought that perhaps a population that might be interested could be students and workers who have not had previous contact with the mental health network." - "I understand it's for a young audience and that's what it aims to represent, today's youth. However, I found some comments a bit uncomfortable, as they could be considered sexist." |
| **Motivation** | - "I think the platform, of course, has utility, that's clear, but I see it a bit like an online course, in the sense that you will sign up and do it because it depends only on you. But if you have an important underlying motivation, in this case, to quit cannabis, it requires a certain dedication, and without a clear motivation, I find it difficult to achieve." - "Yes, totally. In the end, I see it as more of a control tool. In the end, the idea and the intention each person has to reduce or eliminate cannabis use, in whatever ways, are also very important. " - "I believe that without a good foundation, a real motivation to reduce or quit cannabis, or to become more aware, I don't see how support could be a great help or a differentiating factor." | - "Speaking of the wheel of change, I believe this tool would be found in the preparation or action phase, as there is no space for resolving ambivalence, nor do I think it motivates change." - "I think it would be interesting to analyze and help the patient who has not yet reached the action phase or is still in pre-contemplation, and to understand the severity level of each patient. For me, there are two types of users: those who only want to reduce or moderate their use and those whose goal is abstinence. Therefore, by conducting an initial survey/questionnaire to understand the user's profile, we could determine the modules that are best suited to their needs." - "I believe that the platform is very much aimed at those people whose main goal is abstinence. Therefore, if someone's aim is reduction, it may create some resistance, causing them to stop using the website. So, by clearly defining the users' goals, there may be more adherence." |
| **Problem awareness** | - "Additionally, it has helped me identify moments of craving. It helps me calm down because I already know what will happen. Knowing how to identify it and being aware that it will be temporary." - "This explanation of craving is very well done. I had never named what I feel. I would create a more explicit module about the negative consequences of use to make us aware of how bad it is." - "I also see it as a control tool or as a way to name something that you might not be able to control, or aren't really aware of the situation as it truly is. By approaching it in a more realistic and official way, you might realize that there’s something that you're not handling well." - "One of the things I would highlight in its favor is the register, where you state how much you estimate you will smoke next week, and compare it with what you actually smoked. Sometimes we don't consider how much we smoke, and when you state it, you become slightly more aware of the quantity you smoke." - "In the end, the good thing is that you see yourself very represented in certain points: this happens to me, this is my day-to-day (and maybe it shouldn't be), or this has never happened to me, or the day might come when this could happen. It makes you more aware of your problem." - "Maybe you don't see yourself as the typical stoner, but then you realize that you actually are. Well, it makes you reconsider those things." |  |
| **Economic awareness** | - "I find the platform useful but it lacks some “push” in the economic sense. In the end, it also has a significant economic impact; it's not cheap. That is, not just how much you consume, but how much it is costing you. I think this is something that could be further exploited, in order to be aware of how much you're spending on this." - "Since you have to report the amount, I agree that a simple calculation of your monthly expenses could be made to see how much money you've saved by reducing your consumption." |  |
| **Guidance and use** |  |  |
| **Virtual treatment - In favor** | - "I find the anonymity aspect very interesting. When you go to a hospital, in a way, you're putting yourself out there and you could run into anyone. Having that aspect be totally private is very interesting. In fact, there are many people who use cannabis but never disclose it." - “Many of the people who don’t access hospital care are young people who might be a good fit for this because they might not realize the seriousness of their problem. This approach, which could be seen as a less invasive treatment option, might be more appealing to them." | - "I think it's aimed at people who are having their first encounters or who, to some extent, are moderate users." - "The platform seems to target the general population at risk of cannabis abuse, but I see a couple of modules, especially the later ones, that are very focused on people with severe abuse. The platform can be aimed at people having their first contact with cannabis but, at the same time, it can be an additional tool for patients with cannabis use disorder. I liked seeing that it's well adapted to both ends of the consumption spectrum, those with cannabis use disorder and those who use it in a more recreational way." - "I would also like it to be used as a tool to help maintain the changes that have been achieved." |
| **Virtual treatment - Against** | - “Well, I don't see it as a good solution. How does it fit in the end? If the patient seeking help has decided to take the step of looking for more personal support by going to the doctor, offering them online assistance, which is colder and less personal, doesn't make sense. The person who decides to seek in-person support wants exactly that: in-person support.” - "I completely disagree. Online support does nothing for me; I need physical and in-person support. I believe it's vital for a professional to provide in-person support to truly understand the problem, and the platform will never fully achieve that." | - "I find it difficult to imagine a patient with dual pathology or severe dependency using CANreduce." - "While looking at the website, I had a certain fear that people accessing it before attending an in-person consultation might become frustrated if they fail to reduce their use. My fear is that due to the frustration of feeling like they have failed, they may not want to attend public substance abuse treatment centers either. As you mentioned, there are many people who feel ambivalent and display varying levels of motivation, and these aspects are easier to address in therapy than with the platform." - "It wouldn't work as a substitute for in-person therapy. I believe it cannot encompass the full scope of work conducted in face-to-face therapy. I feel that many working areas that the platform could not offer would be left out, such as companionship and support. But it can be an additional tool that the person can benefit from." |
| **Virtual support from Laura - Therapist gender** | - "From a marketing point of view, women are more associated with caregiving; you trust them more when it comes to sharing your secrets, and so on." - "Personally, I'd like to say it wouldn't have influenced me, but subconsciously, this kind of help is very tied to women." |  |
| **Virtual support from Laura - In favor** | - “Maybe if I had had contact with a real person, I would have written to them, and maybe I wouldn’t have given up. Because there’s already someone with a name who exists on the platform.” - "If there had been an open forum, I would have asked some questions, like doubts that might arise." - "I would have preferred it if Laura personally wrote to me to check on how I was doing and how I was feeling." - "I believe you are humanizing the process and, in some way, receiving that human touch helps you progress. Otherwise, you might even become demotivated, feeling like you are alone, interacting with a machine. Knowing you have support makes it warmer, being able to talk to a person or feeling that someone is answering. That could help a person stay on the treatment." - "But obviously, it's good being able to contact someone directly to answer doubts." | - "In addition to this initial questionnaire, it would also be beneficial for the user to receive some kind of feedback. This feedback should not only tell them which phase they are in, but also provide some guidance or motivation to help create a connection." |
| **Virtual support from Laura - Against** | - "I’m old-fashioned when it comes to interacting through social networks and email. If I have a question, I tend to look for the answer elsewhere and try to solve it on my own." - “I don’t need anyone to check on my progress”. - "I see it differently, maybe because I'm more detached in this regard. It's a topic I don't want much input on or to share with many people outside my close circle. Going back to the beginning, I think without a solid foundation, a real motivation to reduce or quit cannabis, or to be more aware, I don't see that the support can be of great help or a differentiating factor, because it's a cold kind of support: it's still online, through messages. You always have the option to ask for help, I think it should be available, but I don't see it as a differentiating value without real motivation." - "But the fact that this support is virtual, to make it more human, it should be in person." |  |
| **Complement to in-person therapy** | - "I think it’s important to emphasize that this can be a starting point for seeking help but, in case it’s not enough, you can turn to in-person resources like the hospital. Maybe I would further complete the transition from one to the other. This could be an aid to start reducing, but if it’s not enough for you, you should be guided to the next phase. I'm not exactly sure how, but that's the idea." - "It’s very useful to combine both options, so that the doctor doesn’t have to explain things that I can read on the platform, and we have time to talk about personal affairs, things that are mine, individual and personalized." - "It can also be very useful in terms of optimizing the doctor's resources. You can skip talking about all the information that's available on the platform. I also find it interesting that the doctor has a record of your cannabis use in the past week, for example." - "As a complement disconnected from the topics you discuss with the doctor, it doesn’t make much sense. It only makes sense if the doctor knows exactly the information you get from there and the data you provide. It should be a complement for people who already go to the hospital for assistance, with this being a secondary resource." | - "For me, it is completely complementary and I think in very few cases could it be a substitute for a therapeutic process. The therapeutic bond and support would be missing." - "Something very interesting to highlight is that there may be patients who cannot access in-person therapy. There are very few professionals specialized in the subject, there are long waiting lists, and appointments are usually very spaced out. In this sense, it could work as an initial form of help and might encourage the person to eventually seek professional in-person assistance." - "I would say that the application would be very useful during the waiting phase." - "That's why I think it should be more focused on being a support tool during treatment or for maintaining the changes that have been achieved, since there will be work done in consultation and the patient will be much more solid." - "I think psychologists and psychiatrists, especially for keeping track of consumption and providing the patient with more information to work on their awareness. I wouldn't extend it to anyone else, although I do think the information on the platform is very good, excellently written, and very accessible." - "I think it could also be useful for social workers and nurses." - "Exactly, it could be very beneficial for all professionals working with these patients. It's a useful tool as a complement to other therapies, and it could even be implemented from primary care when active consumption is detected." - "I believe that in any process involving adherence, the role of a professional is key. I might miss the presence of a professional or the message that they can always connect with their reference substance abuse treatment center or their primary care doctor whenever needed. In other words, just informing them where to go if necessary. I see that this information is not on the platform. The user should feel supported by the healthcare system whenever they need it." |
| **Content and design** |  |  |
| **Application** | - "I used it on my phone and the information was presented in a very small and condensed way." - "I even had to rotate the phone because I couldn't see all the text." - "On the computer, everything is clearer than on the phone. More care should be taken with how things are displayed on mobile devices." - "It would be cooler if it was an app as there could also be daily notifications, for example, on how much you've smoked today. Having a bit more tracking." - "I think an app format could give it much more utility. I once used an app to quit smoking, and it had little information but was individualized. It asked: 'What percentage have you improved in this and that? What benefits do you see since you started reducing? [...] For me, the key would be using an app that can send notifications and provide personalized information." | - "It could be interesting to have it as an app. It could be more convenient and easier to use." |
| **Notifications** | - "I think we're used to receiving notifications." - "I would have ended up removing a daily reminder on my phone, but I would have liked a weekly message like 'How was your week?' to remind me and allow me to log it in my journal." - "A reminder isn't necessary. If I decide not to smoke, then I don't, and I don't need to write anything down. Writing things down doesn't help me." - "Reminders also seem like homework for children." - "You have to actively access it. However, if you received a notification, there would be more adherence to using it." - "The idea of a daily notification or a small reminder, at the very least." | - "There might be higher adherence if the platform sent reminders." - "I would give users the option to choose whether they want to receive notifications or not, as some users may not like receiving feedback." - "I've had an idea regarding integration with the healthcare system. There could be a pop-up while using the platform, something like: 'If you need help, here's what you can do,' or they could leave a message, which could increase the number of inquiries you receive." |
| **Information** | - "I would make all the modules in video format. For example, I used it on my phone and the information appeared very small and condensed. I would prefer videos because I could take notes on what is being said, which is more comfortable for me. It would be like a video tutorial that asks you to do exercises, etc." - "I feel that the modules are a bit dense." - "I liked the part with the characters because I could perfectly identify with one of the profiles. This way, I could also identify people in my environment who are smokers. Not all smokers are the same." - "Good, but could be improved. The modules and the follow-up forms are very dense and require a lot of effort. For example, I had to even rotate my phone because the text wasn't fully visible. Someone who isn't very used to this might get lost. Also, I didn't understand the consumption graph: there were too many numbers, colors, etc. It needs to be made simpler, more user-friendly." - "I would create a more explicit module on the negative consequences of consumption to gain awareness of how bad it is." - "I would add a panic button that quickly showed the pros and cons in a moment of strong cravings. [...] However, it uses a lot of technical jargon, which might alienate some people." - "For me, consumption is associated with stress and anxiety, and I seek calm in cannabis use. Personally, a message that addresses these two issues, rather than the consumption itself, would be quite appealing and would make me feel represented." - "Having the form be a short and simple text would help a lot too, because otherwise it is associated with exams, training, and boredom." - "It would be interesting to have a search function so we could look up information on terms that are of interest to us." - “Something that is quite common and a big problem with cannabis is the widespread belief that cannabis is harmless, minimally harmful, has few adverse effects, etc., etc. Having them listed and explained here and what they can actually imply breaks this perception a bit." | - "The platform is quite informative." - "I believe there can never be too much information, but the content could be more flexible, allowing users to navigate the information that interests them the most. Some sections have too much text; I would recommend making them more visual, as this type of information is easier to remember." - "An interesting proposal would be to use drop-down menus or allowing users to access links to the topics that interest them the most." - "I liked it a lot, in general; it seems very interactive and easy to use, so I wouldn't propose any changes regarding the design." - "I like the format of the information, but we would have to see how users or patients react." - "I would make the option for in-person help more visible, either by including it in a dedicated module (help module or urgent module) or by adding a reminder in each module that the user can seek help from their reference public substance abuse treatment center at any time." - "I think psychologists and psychiatrists, especially for keeping track of consumption and providing the patient with more information to work on their awareness. I wouldn't extend it to anyone else, although I do think the information on the platform is very good, excellently written, and very accessible." |
| **Small achievements and gamification** | - "But not for 20-year-olds because they may not have seriously considered it yet, and the platform is not gamified. It should be made a bit more engaging. Even for me, the forms were a bit boring, and a 20-year-old would lose interest in the program very quickly." - “I read a lot of information all at once and didn't feel like I was absorbing it. Gamification would be very interesting; it could make everything easier." - “It feels like the platform is an educational course more than a treatment. Within a module, not everything should be displayed in the same way. It should transition from one screen to another, progressing as if it were a training session, but combining text with other more visual elements to divert attention a bit more and make it more engaging." - "Small achievements. Notifying the obligation to upload data, but also having small goals could be interesting. Progressing and seeing your evolution in a clear way, like saying, 'I've reached this point, now where do I go from here?'" - "Also, adding encouraging messages. For example, when you enter the platform and you're close to finishing a module, it could say 'you're almost there, keep going!' This would make it more rewarding for the person using it, and it would highlight their progress." | - "One possibility is to make the modules progressive. That is, a user who hasn't done much work yet should only see the first module, so they don't get overwhelmed with information when they might still be ambivalent or doubtful." - "I think it would be better if the successive modules are not unlocked until the first ones are completed." - "I don't agree with the idea of blocking the modules, as this will prioritize the earlier modules and make them the most visited. It could also happen that a user is more interested in module 7 than the previous ones, or it could give the impression that the first modules are much more relevant." - "Additionally, I like that it informs about the pending pages and allows the user to see their progress.” - "There might be more adherence if the platform used reminders, positive reinforcement, greetings, or provided feedback on the user's progress." |
| **Personalized content** | - “There are some modules that I haven't understood and that don't apply to me.” - "I would prefer it to be more personalized. The modules should be created based on who you are. This way, you can identify with what you are reading, it fits your situation, and it becomes more believable for you." - "I would have liked an added space to list the excuses or reasons why I consumed more than I expected." - "It would be interesting to have a search engine so we could look up information on the terms that interest us." - "For me, the key would be the use of an application that can send notifications and provide individualized information." | - "Based on the success terms of the application, it seems to be aimed at abstinence. However, I think it would be interesting to understand each person’s demands, that is, to know what each individual intends regarding their consumption. Possibly, not everyone's success or goal is to quit smoking; some may just want to maintain moderate consumption." - "I think more work needs to be done on the flexibility and adaptability of the platform to the type of user and their goal, like a “pre-diagnosis”. However, I believe the platform should be useful for all types of users." - "In addition to this initial questionnaire, it would also be beneficial for the user to receive some kind of feedback that not only tells them what phase they are in but also provides some sort of guidance or motivation to help create a connection." - "One way to adapt the platform for people with cognitive difficulties is to present the information more simply. For them, it might seem like an overwhelming amount of information or they might have trouble maintaining attention. This type of user would benefit from simplicity." - "If this survey is conducted at the beginning to understand the user's current state, the results of this survey can be used to recommend certain modules over others.” - "Perhaps if the content were tailored to the users' goals and their stage in the change process, there would be greater adherence." |
